# Transcriptome analyses identify 10 deregulated hub genes and essential molecular mechanisms in early-onset colorectal cancer

**DOI:** 10.3389/fonc.2025.1655143

**Published:** 2025-09-29

**Authors:** Hieu Duc Nguyen, Ming-Jenn Chen, Chung-Ying Lee, Yi-Chun Ni, Ke Xin Yee, Yung-Fu Wu, Kuen-Haur Lee

**Affiliations:** ^1^ Graduate Institute of Cancer Biology and Drug Discovery, College of Medical Science and Technology, Taipei Medical University, New Taipei City, Taiwan; ^2^ PhD Program for Cancer Molecular Biology and Drug Discovery, College of Medical Science and Technology, Taipei Medical University, New Taipei City, Taiwan; ^3^ Department of Surgery, Chi Mei Medical Center, Tainan, Taiwan; ^4^ Department of Sports Management, College of Leisure and Recreation Management, Chia Nan University of Pharmacy and Science, Tainan, Taiwan; ^5^ Division of Gastroenterology and Hepatology, Department of Internal Medicine, Taipei Medical University Shuang Ho Hospital Ministry of Health and Welfare, New Taipei City, Taiwan; ^6^ Gastroenterology and Hepatology, Taipei Medical University School of Medicine, Taipei, Taiwan; ^7^ TMU Research Center for Digestive Medicine, Taipei Medical University, Taipei, Taiwan; ^8^ National Defense Medical Center, School of Medicine, Department of Medical Research, Tri-Service General Hospital, Taipei, Taiwan; ^9^ Cancer Center, Wan Fang Hospital, Taipei Medical University, Taipei, Taiwan; ^10^ TMU Research Center of Cancer Translational Medicine, Taipei Medical University, Taipei, Taiwan

**Keywords:** early-onset colorectal cancer, metadata, transcriptome, risk model, EoCRC cell model, cell cycle, apoptosis, EOCRC

## Abstract

**Introduction:**

The rapid increase in early-onset colorectal cancer (EoCRC) case numbers in recent years indicated an urgent need to identify the essential mechanisms and markers for EoCRC diagnosis and treatment. Therefore, this study aimed to analyze the metadata to overcome the limitation of the sample number of previous EoCRC research and to identify central mechanisms and genes that are crucial for EoCRC.

**Methods:**

This study employed statistical analysis of data from the cBioPortal and GEPIA databases to identify overexpressed EoCRC genes. Using a protein–protein interaction map, it identified hub genes. The function of these genes was clarified via risk model, survival, and cell model analysis.

**Results and Discussion:**

The results of clinical data analysis showed an increased rate of late-stage diagnosis and a lower overall survival of the EoCRC cohort. A total of 953 enriched gene samples were detected in EoCRC and 89 genes were identified as EoCRC overexpression genes. From the protein–protein interactions among 53 genes, the top 10 hub genes showed potential for EoCRC diagnosis and prognosis by linking gene expression to diagnosis and survival analysis data. The knockdown of four selected hub genes in the cell model identified the association of EoCRC overexpression hub genes with tumor development and suggested their role in mTOR signaling, cell cycle, and apoptosis regulation. In summary, the study analyzed molecular and clinical data to identify hub genes associated with cancer prognosis in patients with EoCRC. These genes may serve as targets for diagnosis and treatment.

## 1 Introduction

Early-onset colorectal cancer (EoCRC) is used to describe patients who are diagnosed with colorectal cancer before 50 years old; this term contrasts with late-onset colorectal cancer (LoCRC). In earlier studies, because patients with EoCRC only accounted for 12% of total patients with colorectal cancer (CRC) (American Cancer Society, https://www.cancer.org), EoCRC did not receive attention despite the evidence suggesting a low prognosis in younger patients. However, the Surveillance, Epidemiology, and End Results database from 1975 to 2010 showed an unexpected increase of patients in the age range of 20–34 (90% and 124%, respectively) and 35–49 years (28% and 46%, respectively) (1). Another analysis at that time showed a 2% rise per year of EoCRC from 1994, and this gradually turned EoCRC into a worldwide phenomenon (2). Therefore, along with the lowering of the recommended screening age for CRC average-risk adults, the diagnosis, treatment, and mechanism of EoCRC have become increasingly urgent in recent years.

Because of the limitation of EoCRC data, many previous studies used the data of CRC-related early-onset diseases to establish the link of EoCRC to many different risk factors from germline genetics, environment, and life habits (3, 4). Although the results did not show a clear relationship between genetics and EoCRC, the molecular mechanism was still mentioned in the research of Shah et al., which showed the trend of histopathological and molecular feature differences in patients with EoCRC (5). A later study on the ACCENT database and study by Cercek et al. focused more on genetic variance; both studies also showed a high percentage of germline variants in patients under 35 years old (6, 7).

On the other hand, gene expression analysis can also be a potential approach to resolving unanswered questions of previous research (8). The mutation database only showed the initialization of cancer development via the gain function of oncogenes and the loss function of suspended genes (9, 10). By analyzing gene expression levels, the difference in biological pathways between EoCRC and LoCRC can be detected. Gene expression analysis can also be used to detect the biomarker for EoCRC (11).

The study of the gene expression system of EoCRC has only gradually received attention in the past decade, in which the role of the Wnt signaling pathway has been identified in EoCRC samples (12). Analyses on EoCRC also show the age-related role of Myc overexpression along with lncRNA WiNTRLINC1 and the ASCL2 gene (13). The transcriptome of patients with EoCRC from The Cancer Genome Atlas (TCGA) suggested that seven genes had increased expression in younger patients but only PEG10 was associated with poor overall survival (OS) (14). In addition, EoCRC samples also show the relevance of overexpression of genes with the apoptosis/inflammation, adhesion, and proliferation signaling pathways (14, 15). However, most studies are limited by sample size and the availability of patient-matched control samples.

Therefore, this study aims to use the metadata from both TCGA and other available datasets in the cBioPortal database. The analysis of a larger number of EoCRC and LoCRC samples may suggest the molecular pathway and the important marker of EoCRC.

## 2 Materials and methods

### 2.1 Data collection

The available CRC datasets from the cBioPortal database (https://www.cbioportal.org) (**Table 1**) were collected, including clinical, genome transcriptome, and mutation data. In addition, the validation of the cBioPortal results was performed using clinical and gene expression data from TCGA data and the GEO dataset GSE39582 in the NCBI database (https://ncbi.nlm.nih.gov/), which were selected for their large sample sizes to ensure more accurate statistical analyses. The samples from collected data were divided into two groups: the below 50-year-old cohort (EoCRC) and the above 50-year-old cohort (LoCRC).

**Table 1:** Colorectal cancer datasets included in this study.

Dataset name	Number of CRC samples
MSK MetTropism (MSK, *Cell* 2021)	4,555
Colorectal Adenocarcinoma (MSK, 2022)	166
Colorectal Adenocarcinoma (DFCI, *Cell Reports* 2016)	619
Colorectal Adenocarcinoma (Genentech, *Nature* 2012)	74
Colorectal Adenocarcinoma (TCGA, Firehose Legacy)	640
Colorectal Adenocarcinoma Triplets (MSK, *Genome Biol* 2014)	138
Colorectal Cancer (MSK, 2022)	22
Colorectal Cancer (MSK, *Gastroenterology* 2020)	471
Colorectal Cancer (MSK, *JCO Precis Oncol* 2022)	47
Disparities in metastatic colorectal cancer between Africans and Americans (MSK, 2020)	64
Metastatic Colorectal Cancer (MSK, *Cancer Cell* 2018)	1,134
Rectal Cancer (MSK, *Nature Medicine* 2019)	339
Colon Adenocarcinoma (CaseCCC, *PNAS* 2015)	29
Colon Cancer (CPTAC-2 Prospective, *Cell* 2019)	110
Total	8,408

### 2.2 EoCRC identification

To identify EoCRC overexpression genes, a two-step analysis was conducted. First, gene expression profiles were statistically compared based on sample-type category. The comparison between EoCRC and LoCRC was sequentially performed on primary and metastatic colorectal cancer samples, respectively, using the cBioPortal group comparison tool. From each of these comparisons, amplification (AMP) genes significantly enriched (*p* ≤ 0.05) in EoCRC samples were selected. Then, a screening step was made between the enriched gene list and the CRC overexpressed gene list (logFC ≥ 1, *p* ≤ 0.05) from the GEPIA database (http://gepia.cancer-pku.cn). The overlapping genes were known as EoCRC overexpressed genes.

### 2.3 Protein–protein interaction analysis and hub gene identification

The EoCRC overexpressed genes were used to construct the protein–protein interaction (PPI) network by the STRING database (https://string-db.org/). The top 10 hub genes based on their degree were identified by the Cytoscape/CytoHubba package (16). After that, hub gene interactions were validated with the Genemania database (https://genemania.org/). The gene correlation was confirmed by the GEPIA database.

### 2.4 Biological pathway analysis

MSigDB Hallmark analysis was performed on EoCRC overexpressed genes using the Enrichr database (https://maayanlab.cloud/Enrichr/). The corelation gene pathway of selected hub genes was analyzed using Ingenuity Pathway Analysis (IPA) (17). Visualization of the top 10 pathways was performed on the imageGP website (http://www.ehbio.com/ImageGP).

### 2.5 Multivariate and risk model analysis

Using the Survival R package, we assessed the relationship between clinical data—including gender, clinical tumor stage (Stage), stage describing size of the original primary tumor (T stage), and stage describing the number of lymph nodes (N stage)—and cohort survival. The data were then subsequently used to perform a multivariate Cox regression analysis, with the findings presented in a forest plot.

To construct a risk model analysis, gene expression data from the TCGA and GSE39582 datasets were also analyzed using multivariate Cox regression to calculate coefficient values. The risk score for all samples was calculated using the following formula: risk score = ∑coefficient value ∗ expression level. Based on the median value of the risk score, the samples were divided into high-risk and low-risk groups. The log-rank test was performed to compare the effect of gene expression on OS between the two groups.

### 2.6 Cell line and siRNA transfection

The age-related cell model was built using four human colon cancer cell lines (HT29, HCT116, LOVO, and HCT15) that were derived from donors aged 44–67 years who were selected for this study. The CRL1459 was used as the healthy colorectal cell line. All cells were cultured in RPMI medium at 37°C and 5% CO_2_, which was added to 1% streptomycin and penicillin and 10% fetal bovine serum (FBS) (Corning).

The hub genes that exhibited a strong correlation with our age-related cell model were selected for further functional analysis. To build the hub gene targeted knockdown EoCRC cell model, the siRNA for four genes—GMNN, KPNA2, MYC, and PRDX4 (**Table 2**)—was designed and synthesized by BiONEER (Korea) and transfected into HCT116 cells using INTERFERin^®^ *in vitro* siRNA/miRNA transfection reagent (Polyplus) for 48 h, following the company’s guidelines. The mRNA efficiency of the siRNA was evaluated using reverse transcription-quantitative polymerase chain reaction (RT-qPCR).

**Table 2:** siRNA list.

siRNA name	Sense sequence	Antisense sequence	Efficiency (%)	SD
KPNA2 siRNA1	CUGUAGAGGAAGAGGAAGA	UCUUCCUCUUCCUCUACAG	53.86	0.03
KPNA2 siRNA2	GAUUCAAGAACAAGGGAAA	UUUCCCUUGUUCUUGAAU	44.86	0.08
PRDX4 siRNA1	CUGGAAACCUGGUAGUGAA	UUCACUACCAGGUUUCCAG	67.54	0.05
PRDX4 siRNA2	CUCUGAAUGAUCUUCCUGU	ACAGGAAGAUCAUUCAGAG	60.24	0.03
MYC siRNA1	GUCAGAGUCCUGAGACAGA	UCUGUCUCAGGACUCUGAC	52.99	0.07
MYC siRNA2	GUCACCAUCUUGACUCCUA	UAGGAGUCAAGAUGGUGAC	33.82	0.03
GMNN siRNA1	GUAUAUGGCAGAGCUAAUA	UAUUAGCUCUGCCAUAUAC	65.23	0.08
GMNN siRNA2	GAAGAAACUGUUGAGGAUU	AAUCCUCAACAGUUUCUUC	58.80	0.04

### 2.7 RNA extraction, cDNA synthesis, and reverse transcription-quantitative PCR

Total RNA was extracted from siRNA-transfected cells using GENEzol™ RNA extraction (Geneaid) and converted into cDNA using MIIIs™ 1st Strand gDNA clear cDNA Synthesis SuperMix (BIONOVAS). Primers were designed using Primer−BLAST (National Center for Biotechnology Information) (**Table 3**). GAPDH was used as the internal control gene. qPCR was performed using the Lightcycle 96 system (Roche) in 40 cycles with the QuantiTect SYBR Green PCR Kit (QIAGEN), 1 pmol/µL forward and reverse primer, 10 ng/µL cDNA template, and PCR-grade water. The qPCR conditions included an initial denaturation step for 10 min at 95˚C, followed by 40 cycles of denaturation at 95˚C for 15 s, and annealing/extension at 55˚C for 45 s.

**Table 3:** Primer list.

Gene name	Forward primer	Reverse primer	Product length (bp)
GAPDH	GTCTCCTCTGACTTCAACAGCG	ACCACCCTGTTGCTGTAGCCAA	131
GMNN	AAACGGAGAAAGGCGCTGTA	CAGGCGGGCAATTTCATTGT	90
KPNA2	AGGCTGTGGTAGATGGAGGT	AGAGCCCAGACAGCTTGTTC	91
MYC	GCCAAGAGGGTCAAGTTGGA	CGTTGTGTGTTCGCCTCTTG	118
PRDX4	CGCTTTTGGCGACAGACTTG	GCCCAAGTCCTCCTTGTCTT	125

### 2.8 Western blot analysis

The total transfected cell mass including suspension death cell was lysed using RIPA buffer (Invitrogen; Thermo Fisher Scientific, Inc.) supplemented with protease inhibitor (Roche) and phosphatase inhibitor (Roche). The collected protein was adjusted to equal amounts of protein samples and separated by 6%–12% SDS-PAGE. The separated proteins were then transferred onto nitrocellulose membranes (Immobilon, Merck Millipore, Darmstadt, Germany). Specific antibodies (Cell Signaling Technology) for mTOR, caspase 3, PARP, CDK4, and Cyclin D1 were used to detect the presence of mTOR, cell cycle, and cell death marker. Actin was used as the internal control gene. The signal generated by the complex of target protein, primary antibody, and secondary antibody was quantified using the ImageQuant LAS 4000 mini system (GE Healthcare, Chicago, IL, USA). The protein expression levels were further analyzed using ImageJ software (18).

### 2.9 Cell proliferation analysis, cell cycle, and apoptosis assay

To analyze cell proliferation, transfected cells were seeded at a density of 1 × 10³ cells per well in 96-well plates. Cell viability was assessed at 24, 48, 72, and 96 h using the MTT assay. Absorbance was measured at 570 nm using a spectrometer. For cell cycle and apoptosis analysis, the total population of transfected cells was harvested and processed according to the protocols of the Muse^®^ Cell Cycle Kit and the Annexin V & Dead Cell Kit (Cytek Biosciences). Flow cytometry analysis was then performed using the Muse^®^ Cell Analyzer.

### 2.10 Statistical analysis

The comparison of AMP genes between the EoCRC and LoCRC cohorts was performed using the GISTIC algorithm available on cBioPortal (19). Additionally, the CRC overexpression genes between the TCGA CRC and control group were identified using the LIMMA method from GEPIA (20). The cBioPortal clinical data and expression data from the cell model were analyzed using GraphPad Prism 5 software, employing chi-square and *t*-test methods, respectively. For the validation of the results, an *in vitro* triple replication experiment was conducted and statistically analyzed using *t*-test. Statistical significance was indicated by the following levels: *p ≤ 0.05; **p ≤ 0.01; ***p ≤ 0.001; ****p ≤ 0.0001.

## 3 Results

### 3.1 Impact of EoCRC on clinical features

**Figure 1:**
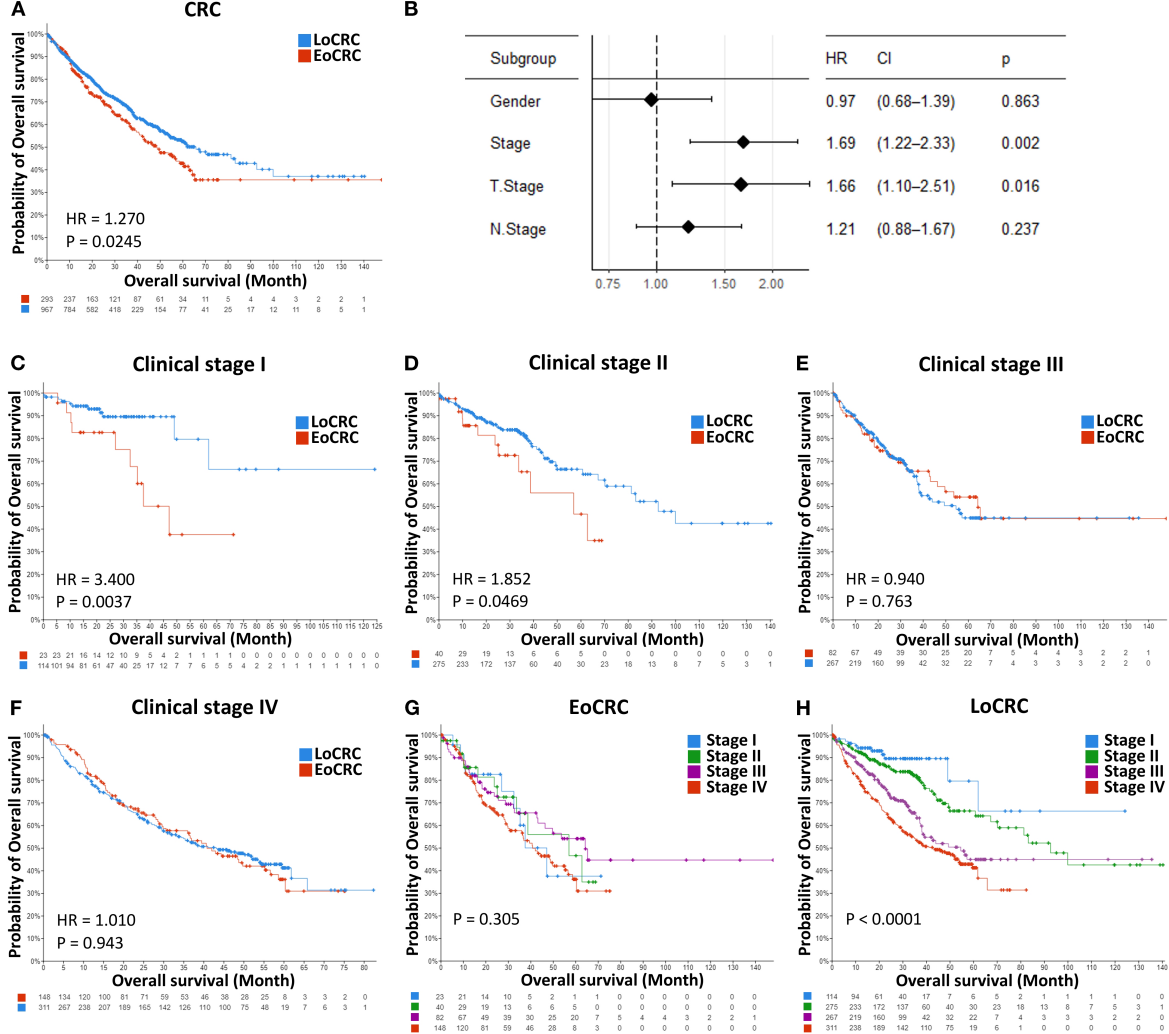
Independent survival analysis between age and clinical stage. **(A)** Comparison of overall survival between the EoCRC and LoCRC cohort. **(B)** Multivariate Cox regression forest plot analysis of total CRC samples available in the cBioPortal database. **(C–F)** Comparison of overall survival between patients with EoCRC and those with LoCRC within each clinical stage (I, II, III, and IV). **(G, H)** Comparison of overall survival across clinical stages (I, II, III, and IV) in EoCRC and LoCRC cohorts, respectively.

A total of 8,408 CRC patient data points were collected from 15 cBioPortal datasets, but only 5,338 samples included patient age information, comprising 1,508 EoCRC and 3,830 LoCRC samples (**Table 4**). The statistical analysis indicated that the rate of patients with EoCRC diagnosed at a late clinical tumor stage and N stage was significantly higher than that in the LoCRC group (*p* < 0.0001). Then, the clinical data of these samples were used to conduct an independent survival analysis between age and clinical stage, which revealed that OS was shorter in the EoCRC group than in the LoCRC group (**Figure 1A**). The multivariate Cox regression forest plot analysis confirmed that a higher rate of late clinical tumor stage negatively impacts patient survival (**Figure 1B**). To demonstrate that the shorter survival time of the EoCRC group is not solely attributed to late medical examination, we compared OS between patients with EoCRC and LoCRC at each stage. The results showed that the EoCRC cohort had significantly lower OS than the LoCRC cohort in the early stages (stages I and II) (**Figures 1C, D**) but not in the late stages (stages III and IV) (**Figures 1E, F**). The prognosis of patients with EoCRC was nearly the same regardless of whether the cancer was detected early or late (*p* = 0.304) (**Figure 1G**), rather than depending on stage like the LoCRC cohort (*p* < 0.0001) (**Figure 1H**). This suggested that the patients with EoCRC did not have a better survival prognosis even when the disease was detected before it progressed to stage III.

**Table 4:** Characteristics of patients with EoCRC and LoCRC according to colorectal cancer status.

Group	EoCRC		LoCRC		*p*-value
	*n*	%	*n*	%	
Number of patients	1,508		3,830		
Gender					
Female	696	46.15	1,869	48.80	0.0816
Male	812	53.85	1,961	51.20	
Clinical stage					
I	28	4.79	287	14.82	<0.0001*
II	65	11.11	543	28.05	
III	169	28.89	524	27.07	
IV	323	55.21	582	30.06	
Unknown	923	–	1,894	–
T stage					
T1	3	3.19	17	2.64	0.3075
T2	11	11.70	113	17.55	
T3	65	69.15	444	68.94	
T4	15	15.96	70	10.87	
Unknown	1,414	–	3,186	–
N stage					
N0	36	37.89	377	58.72	0.002*
N1	33	34.74	156	24.30	
N2	26	27.37	109	16.98	
Unknown	1,413	–	1,467	–
Sample type					
Primary	865	59.13	2,008	65.11	0.0005*
Metastasis	598	40.87	1,076	34.89	
Unknown	45	–	746	–

*Statistical significance; *p* ≤ 0.05.

### 3.2 Transcriptome comparison and identification of EoCRC hub genes

**Figure 2:**
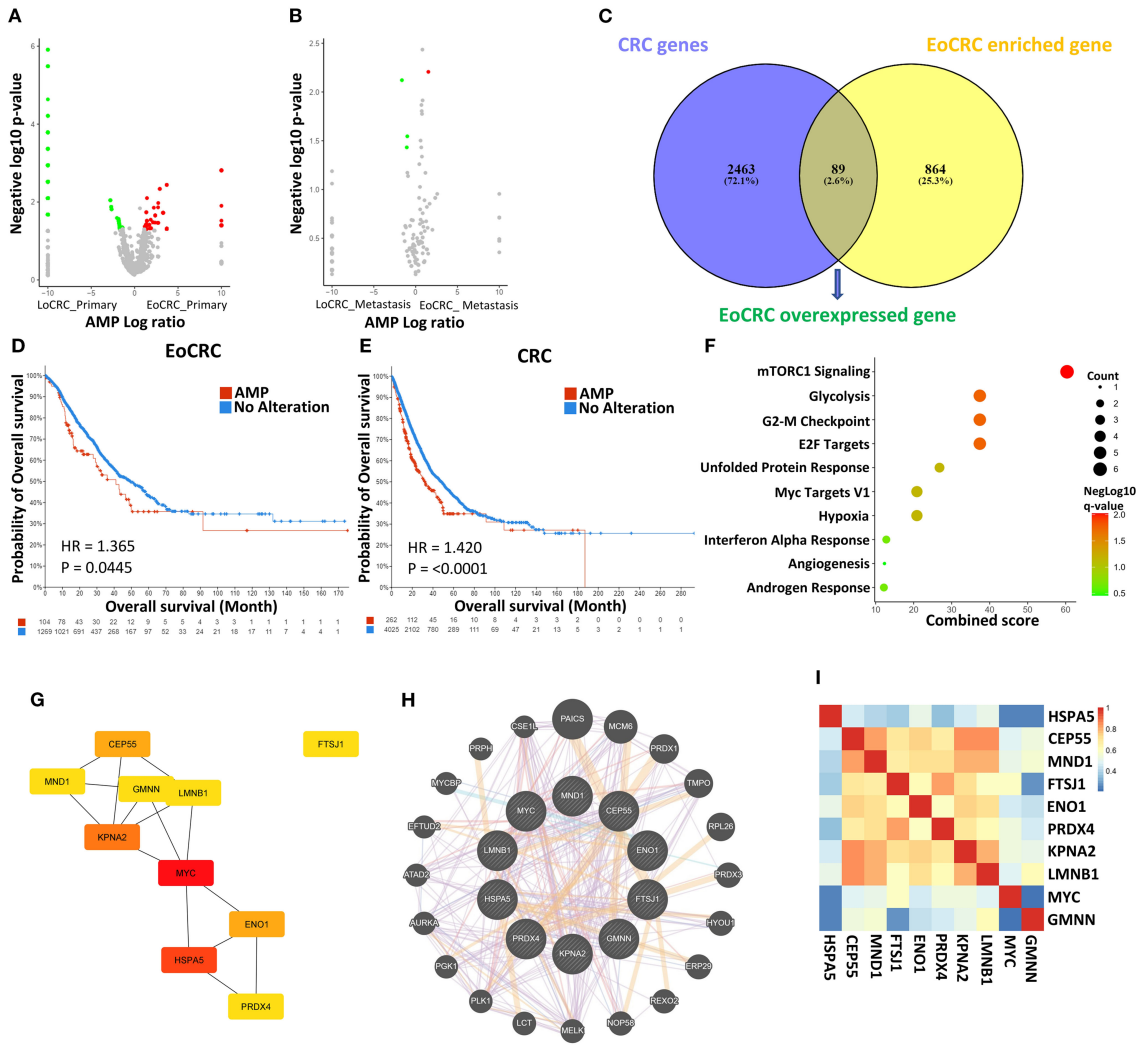
Analysis of gene expression data in EoCRC and LoCRC cohorts. **(A, B)** Volcano plots illustrating differentially expressed genes between EoCRC and LoCRC in primary colorectal cancer samples and metastatic samples, respectively. **(C)** Identification of EoCRC overexpressed genes by overlapping EoCRC-enriched genes with CRC gene database. **(D, E)** Comparison of overall survival between patients with amplified (AMP) and no-alteration (No Alteration) status of EoCRC overexpressed genes in the EoCRC and general CRC cohorts, respectively. **(F)** MSigDB Hallmark Pathway analysis of EoCRC overexpressed genes. **(G)** Identification of the top 10 hub genes based on their degree, using the Cytoscape/CytoHubba package. **(H)** Confirmation of protein–protein interactions (PPIs) among hub genes via GeneMANIA. **(I)** Validation of gene correlation among hub genes using GEPIA.

By comparison of the AMP gene between EoCRC and LoCRC, the difference in transcriptome between the two groups was investigated. The results of the analysis showed a higher number of significant genes (*p* ≤ 0.05) in the primary tumor samples (**Figure 2A**) than in the metastasis tumor samples (**Figure 2B**), a finding that is consistent with the distinct clinical outcomes observed in patients with early-stage EoCRC. A total of 953 genes that were enriched in the EoCRC patient group were selected and screened through the CRC overexpression gene list obtained from the GEPIA dataset to identify key genes with a direct impact on tumor progression (**Figure 2C**). The overexpression of 89 overlap genes showed a strong impact on both patients with EoCRC and all patients with CRC (**Figures 2D, E**). Through the prediction of signaling pathways, these genes primarily participate in mTORC1 signaling pathways (*p* = 0.009), followed by glycolysis (*p* = 0.018), G2-M checkpoint (*p* = 0.018), and E2F targets pathway (*p* = 0.018) (**Figure 2F**).

The STRING tool was used to predict PPI between the genes in the list. The results showed that 53 genes formed a PPI network (**Supplementary Figure S1A**). The top 10 hub gene-coded proteins showed a central role in the map (**Figure 2G**). These hub genes were known by the UniProtKB/Swiss-Prot Function as important for cell development (**Supplementary Table S1**). The interaction between these genes was confirmed through the Genemania tool (**Figure 2H**), and the prediction results also suggested that 22 other potential genes may be the signaling intermediaries for these genes. Correlations were also confirmed by the GEPIA dataset, showing that all are positive interactions (*p* ≤ 0.05) (**Figure 2I**).

### 3.3 Using a risk model as a validation of the hub genes’ impact

**Figure 3:**
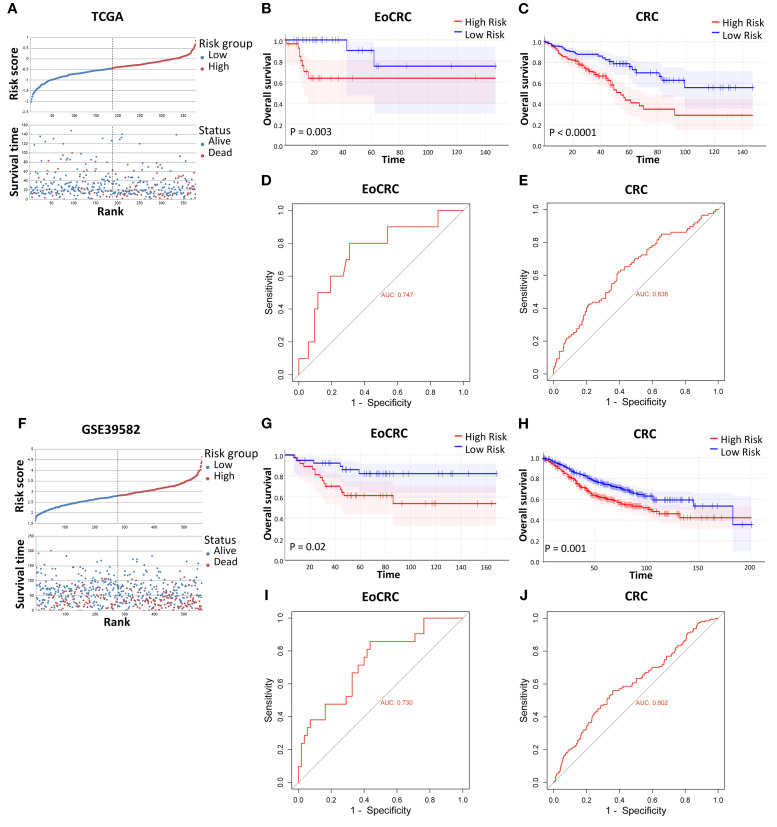
Validation of risk models on individual CRC datasets. **(A, F)** Relative survival visualization of CRC risk groups, separated by risk score median, in the TCGA and GSE39582 datasets, respectively. **(B, C, G, H)** Comparison of overall survival between high-risk and low-risk groups in the TCGA EoCRC and CRC cohorts, and the GSE39582 EoCRC and CRC cohorts, respectively. **(D, E, I, J)** ROC curve analysis evaluating the predictive performance of the risk model in the TCGA EoCRC and CRC cohorts, and the GSE39582 EoCRC and CRC cohorts, respectively.

Because of the strong effects of high expression of hub genes in the OS of patients with EoCRC and CRC (**Supplementary Figure S1B, C**) from cBioPortal datasets, the impact of these hub genes was validated through the risk model in TCGA and GSE39582 datasets. We divided patients in each dataset into two risk groups based on the median risk value calculated (**Figures 3A, F**). The results confirmed that the high-risk group, as indicated by the expression of hub genes in both CRC and EoCRC patient groups, had significantly poorer survival rates compared to the low-risk group. This finding was consistent across both TCGA (**Figures 3B, C**) and GSE39582 datasets (**Figures 3G, H**). Although the predictive performance remains at a poor level, the AUC demonstrated higher diagnostic performance in the EoCRC cohort (0.747 and 0.730 in the TCGA and GSE39582 datasets, respectively) (**Figures 3D, I**), suggesting a more important role of hub genes in EoCRC compared to LoCRC (**Figures 3E, J**).

### 3.4 Using a cell model as a validation of the hub genes’ impact

**Figure 4:**
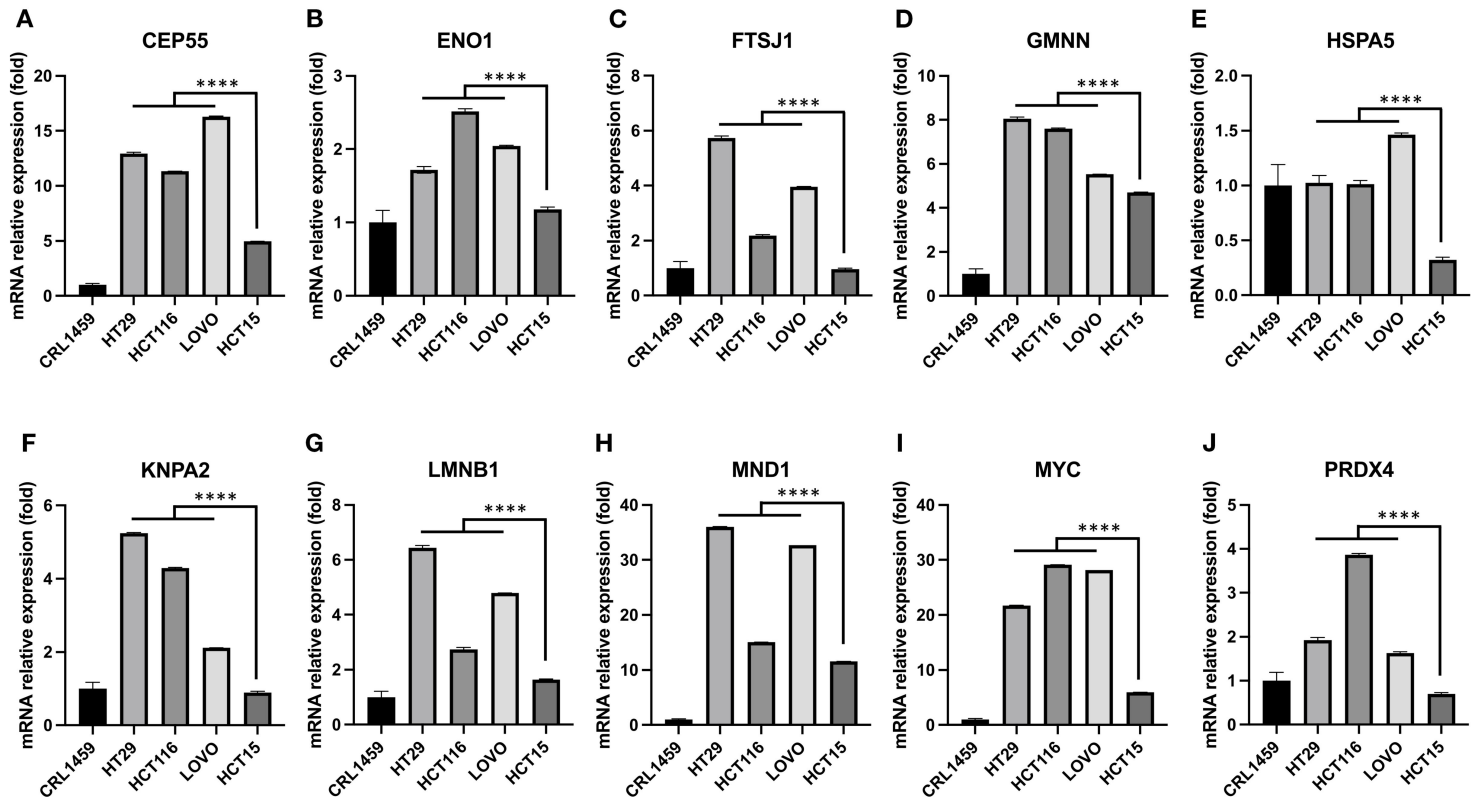
The relationship between hub gene expression levels and the age of cell donors in CRC cell lines. Hub gene expression was measured in CRC cell lines arranged from younger to older donor age—HT29 (44 y), HCT116 (48 y), LOVO (56 y), and HCT15 (67 y)—and compared with the normal colorectal cell line CRL1459. Overall, CRC cells showed higher expression than control for most hub genes, except HSPA5 **(E)**. An inverse relationship with donor age was evident for ENO1 **(B)**, GMNN **(D)**, KPNA2 **(F)**, MYC **(I)**, and PRDX4 **(J)**. For the remaining genes **(A, C, E, G,** and **H)**, significant differences were still observed between cell groups derived from EoCRC and LoCRC donors, with LOVO (56 y) serving as a clear dividing point, though the age-related trend was less stable. Based on stability and significance across lines, GMNN, KPNA2, MYC, and PRDX4 were prioritized for subsequent functional studies in the EoCRC cell model. **** p ≤ 0.0001.

The expression levels of hub genes were assessed in our available CRC cell lines (**Figure 4**). These cells were organized in ascending order of donor age, including HT29 (44 years), HCT116 (48 years), LOVO (56 years), and HCT15 (67 years). The results indicated that these genes displayed higher expression levels compared to the control colorectal cell line CRL1459 except HSPA5 (**Figure 4E**). Additionally, we observed an inverse relationship between gene expression levels and age. This trend was more pronounced for the genes ENO1 (**Figure 4B**), GMNN (**Figure 4D**), KPNA2 (**Figure 4F**), MYC (**Figure 4I**), and PRDX4 (**Figure 4J**). As for the remaining genes (**Figures 4A, C, E, G, H**), they still demonstrated statistically significant differences between the cell groups obtained from EoCRC and LoCRC donors with the LOVO cell being a clear dividing point, but the trend exhibited lower stability. A summary of results showed that GMNN, KPNA2, MYC, and PRDX4 were deemed more stable for further functional analysis in the EoCRC cell model.

### 3.5 Role of EoCRC hub genes on tumor cell proliferation

**Figure 5:**
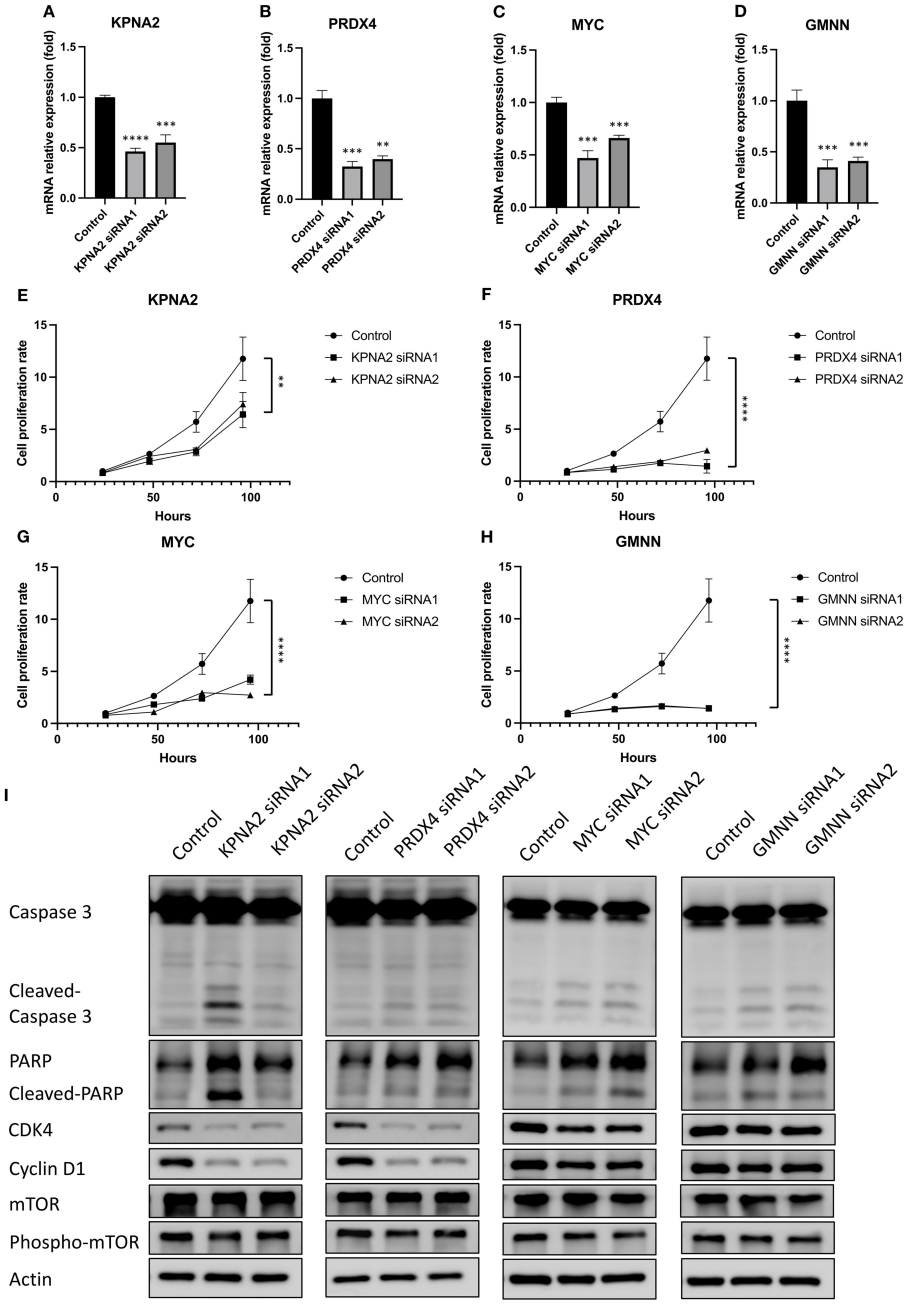
The effect of hub genes on the cell proliferation and cell cycle marker of EoCRC cell line HCT116. **(A–D)** RT-qPCR analysis demonstrating the knockdown efficiency of four selected hub genes. **(E–H)** Assessment of HCT116 cell line proliferation following knockdown of individual hub genes. **(I)** Western blot analysis of protein lysates from hub gene knockdown cell lines, evaluating changes in cell cycle and cell death markers. **p ≤ 0.01; ***p ≤ 0.001; ****p ≤ 0.0001.

Owing to their crucial role in regulating the mTORC1 signal pathway, the EoCRC hub gene function was essential for the development of EoCRC. Mutants of these hub genes were found to hinder both EoCRC and CRC development (**Supplementary Figures S1D, E**). Furthermore, analyzing the co-expressed genes of the chosen hub genes using IPA (**Supplementary Figures S2A–D**) revealed that these genes may play significant roles in cell cycle checkpoints and mitotic regulation (**Supplementary Figure S2E**). To determine the specific role of each gene in EoCRC, a hub gene-targeted knockdown assay was conducted. HCT116 and HT29 were selected due to their high and stable expression of the four genes, but only HCT116 demonstrated high efficiency of siRNA (**Figures 5A–D** and **Table 2**) and was used for further analysis. The results showed that all genes significantly impacted the growth of cancer cells (**Figures 5E–H** and **Supplementary Figures S3A–D**). They also exhibited a strong influence on markers representing the G0/G1 cell cycle checkpoint (CDK4 and Cyclin D1) and cell death (Cleaved-Caspase 3 and Cleaved-PARP) (**Figure 5I** and **Supplementary Figures S3E–H**). Moreover, the gene-targeted knockdown slightly impacted the phosphorylation of the mTOR protein, the pathway of which was related to the EoCRC PPI network (**Figure 2F**) and controlled the G0/G1 phase of the cell cycle (21).

### 3.6 Role of EoCRC hub genes on tumor cell cycle and apoptosis

**Figure 6:**
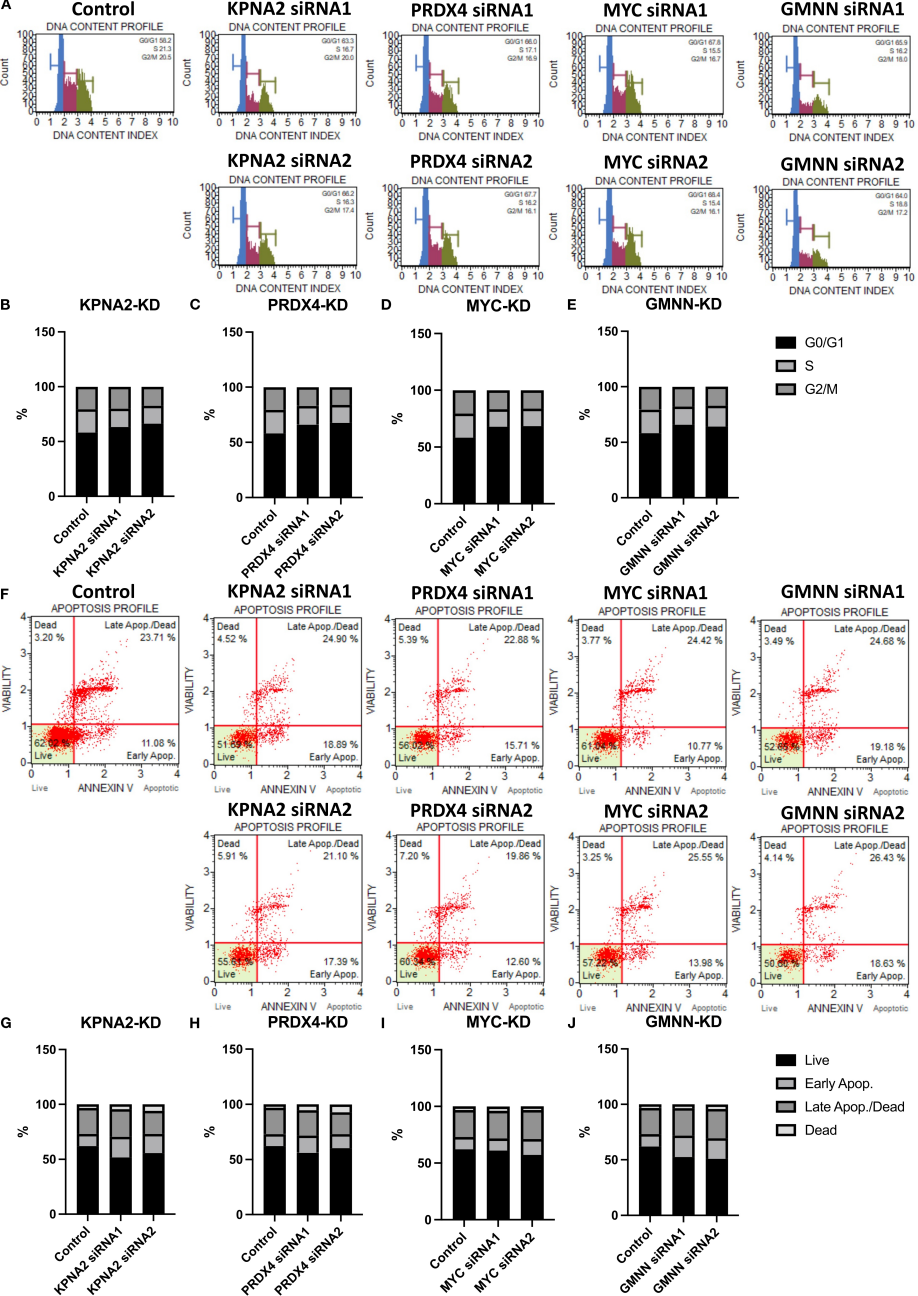
The effect of hub genes on the cell cycle and apoptosis of EoCRC cell line HCT116. **(A)** Cell cycle analysis of HCT116 cell lines following knockdown of hub genes. **(B–E)** Graphs illustrating the percentages of cells in different cell cycle phases for control and hub gene knockdown cell lines. **(F)** Apoptosis analysis of HCT116 cell lines after hub gene knockdown. **(G–J)** Graphs illustrating the percentages of apoptotic cells for control and hub gene knockdown cell lines.

The expression of cell cycle and cell apoptosis markers and the previous functional predictions provided evidence for us to test the role of these genes in cell cycle regulation and apoptosis (**Figures 6A, F**). Following flow cytometry analysis, cells with reduced expression of hub genes tended to arrest in the G0 phase instead of proceeding to the next growth phase (**Figures 6B–E**). In addition, the reduced expression of these genes also directly affected the rate of cells entering apoptosis (**Figures 6G–J**).

## 4 Discussion

Previous studies have shown that the risk of developing EoCRC is associated with genetic, environmental, lifestyle, and late-stage diagnosis factors (3, 4, 22). However, using a large sample size of metadata to avoid biases due to race, genetics, and lifestyle of individual studies conducted in different regions (23–25), this study showed clinical evidence of increased tumor progression in early-onset patients reported in the previous research (26). These progressions not only were due to late detection (27) but also involved a mechanism that causes early-stage tumors to grow more rapidly.

Transcriptomic analysis revealed the significance of 89 overexpressed genes in primary colorectal cancer tumors. These genes play a crucial role in the survival of patients with CRC, particularly those with EoCRC, who often exhibit higher gene expression levels. The primary signaling pathways of these genes are all essential for regulating cell cycle and apoptosis (28, 29). This can be mediated by mTOR and regulated by E2F targeting (21, 30). Furthermore, there was a strong regulatory linkage between mTORC1 signaling and the glycolysis pathway, which is also being investigated. This evidence suggested that these pathways play a role in the connection between cancer and metabolic diseases such as obesity and diabetes (31, 32).

Furthermore, this study identified 10 hub genes that depended on PPI networks. Hub genes were genes with the highest number of interactions with other genes in the network. Therefore, these genes can be regulators or targets mediating various cancer regulatory processes (33, 34). This makes them important for cancer development processes. As regards all 10 genes found in this study and the correlation between their overexpression and the development of patients with CRC, patients exhibiting high expression of these genes are more likely to develop cancer than others in the group (35–38). However, diagnostic results in the EoCRC cohort demonstrated even better performance when utilizing hub genes for diagnosis among all patients with CRC. This has been validated not only in our metadata but also in independent datasets, including TCGA and two GEO datasets. Consequently, these genes may be regarded as potential targets for treatment and diagnosis, particularly for patients with EoCRC, and may play crucial roles in tumor growth.

To further elucidate the potential mechanisms of hub genes in EoCRC, we established a cancer cell model derived from patients of varying ages, reflecting differences in donor origin regarding the gene expression of the cell lines (39). While some genes in cell lines may display different expression results compared to their primary tissues (40)—an observation noted in long-term cultured cell lines—the trend in hub gene expression exhibited a negative correlation with donor age. We analyzed the results from the cell model tests to identify the four genes showing the highest correlation. These genes were expected to be the most affected by aging among those evaluated in this study, allowing the cell model testing to clarify their functions. In a previous study, all four genes were found to be related to cancer. MYC, a proto-oncogene, is frequently amplified or overexpressed in human cancers. It acts as a central transcriptional regulator, promoting cell proliferation and survival (41). KPNA2, a nuclear import protein, is often found elevated in various cancers. Its mislocalization of key regulatory proteins contributes to tumor progression and is linked to poor prognosis (42). PRDX4, an endoplasmic reticulum-resident antioxidant enzyme, helps cancer cells combat oxidative stress, promoting tumor growth, drug resistance, and metastasis (43). GMNN, a DNA replication inhibitor, is frequently overexpressed in tumors. Its dysregulation contributes to uncontrolled cell proliferation and genomic instability, making it a driver of cancer development (44). Both statistical analyses of the impact of the selected hub gene mutations and evaluations of gene knockdown demonstrated significant inhibition of tumor growth.

The EoCRC overexpression gene pathway prediction indicated their involvement in the mTORC1 pathway. Functional predictions and subsequent testing with protein markers in cell models confirmed that all four hub genes play crucial roles in regulating the G0/G1 phase of the cell cycle and apoptosis in EoCRC, which is one of the most significant functions of the mTOR and mTORC1 pathways (21, 45). Notably, there was a complex regulatory relationship between mTOR and MYC (44), and KPNA2 was also known to be regulated by mTOR (46). In this study, we discovered that the feedback impact of KPNA2 and MYC on mTOR phosphorylation may occur through the MYC nuclear transition of KPNA2 (42) or via EoCRC PPI networks. Additionally, we also identified the regulation of mTOR phosphorylation by two genes, PRDX4 and GMNN, suggesting that mTOR may mediate the cell cycle regulation of the selected hub genes. Flow cytometry results revealed that knockdown cells tend to arrest in the G0 phase before progressing to apoptosis. The cell cycle has long been regarded as a pathway through which aging influences cancer cell development (47); however, this study suggests a cell cycle-targeted mechanism by which tumor risk occurs in younger individuals rather than increasing with age (48).

In summary, this study analyzed molecular and clinical data to propose distinct mechanisms in patients with EoCRC associated with cancer prognosis. The identified top 10 hub genes showed an association with initial tumor development, and their roles in cell cycle and apoptosis have been suggested. These genes may serve as potential candidates for further studies to identify possible targets for EoCRC diagnosis and treatment.

## Data Availability

The original contributions presented in the study are included in the article/**Supplementary Material**. Further inquiries can be directed to the corresponding authors.

## Supplementary material

Supplementary Table 1 The top 10 Degree hub genes were identified by Cytoscape/CytoHubba package.

Supplementary Figure 1 Protein-protein interaction (PPI) of EoCRC overexpression genes. **(A)** Protein-protein interaction (PPI) map of EoCRC overexpression genes analyzed by STRING. **(B, C)** Comparison of overall survival between AMP and No Alteration hub genes in EoCRC and CRC cohort, respectively. **(D, E)** Comparison of overall survival between Wildtype and Mutation hub genes in EoCRC and CRC cohort, respectively.

Supplementary Figure 2 Hub gene function pathway prediction and comparison analysis using correlation genes in TCGA EoCRC cohort. **(A–D)** Ingenuity Pathway Analysis (IPA) of four selected hub genes, illustrating their predicted functional pathways. **(E)** Pathway comparison analysis highlighting the shared biological pathways among these hub genes.

Supplementary Figure 3 Statistical analysis of the effect of Hub Genes on the cell proliferation and cell cycle markers of EoCRC cell line HCT116. **(A–D)** Cell viability comparison between control and knockdown HCT116 cell lines, assessed every 24 h after knockdown for each of the four hub genes. **(E–H)** Quantitative protein expression levels of cell cycle and cell death markers for each knockdown cell lines, analyzed using ImageJ software. ns: non-statistical significance; **p* ≤ 0.05; ***p* ≤ 0.01; ****p* ≤ 0.001; *****p* ≤ 0.0001.
