# Supplementary material for: Transcriptome analyses identify 10 deregulated hub genes and essential molecular mechanisms in early-onset colorectal cancer
Source: Front Oncol. 2025 Sep 29;15:1655143. doi: 10.3389/fonc.2025.1655143 (PMC12515628; doi:10.3389/fonc.2025.1655143)
Supplement: Supplementary Table 1 — The top 10 Degree hub genes were identified by Cytoscape/CytoHubba package. [file DataSheet1.docx]

**Supplementary Table 1.** The top 10 Degree hub genes were identified by Cytoscape/CytoHubba package

| **Gene symbol** | **Degree** | **Protein name** | **UniProtKB/Swiss-Prot Function** |
| --- | --- | --- | --- |
| HSPA5 | 10 | Heat shock protein family A (Hsp70) member 5 | Endoplasmic reticulum chaperone that plays a key role in protein folding and quality control in the endoplasmic reticulum lumen. |
| CEP55 | 5 | Centrosomal protein 55 | Plays a role in mitotic exit and cytokinesis. |
| MND1 | 4 | Meiotic nuclear divisions 1 | Required for proper homologous chromosome pairing and efficient cross-over and intragenic recombination during meiosis. |
| FTSJ1 | 4 | FtsJ RNA 2'-O- methyltransferase 1 | Methylates the 2'-O-ribose of nucleotides at positions 32 and 34 of the tRNA anticodon loop of substrate tRNAs. |
| ENO1 | 5 | Enolase 1 | Glycolytic enzyme the catalyzes the conversion of 2-phosphoglycerate to phosphoenolpyruvate. |
| PRDX4 | 4 | Peroxiredoxin 4 | Thiol-specific peroxidase that catalyzes the reduction of hydrogen peroxide and organic hydroperoxides to water and alcohols, respectively. |
| KPNA2 | 6 | Karyopherin subunit alpha 2 | Functions in nuclear protein import as an adapter protein for nuclear receptor KPNB1. |
| LMNB1 | 4 | Lamin B1 | Lamins are components of the nuclear lamina, a fibrous layer on the nucleoplasmic side of the inner nuclear membrane, which is thought to provide a framework for the nuclear envelope and may also interact with chromatin. |
| MYC | 12 | MYC proto-oncogene, bHLH transcription factor | Transcription factor that binds DNA in a non-specific manner, yet also specifically recognizes the core sequence 5'-CAC[GA]TG-3' |
| GMNN | 4 | Geminin DNA replication inhibitor | Inhibits DNA replication by preventing the incorporation of MCM complex into pre-replication complex (pre-RC). |


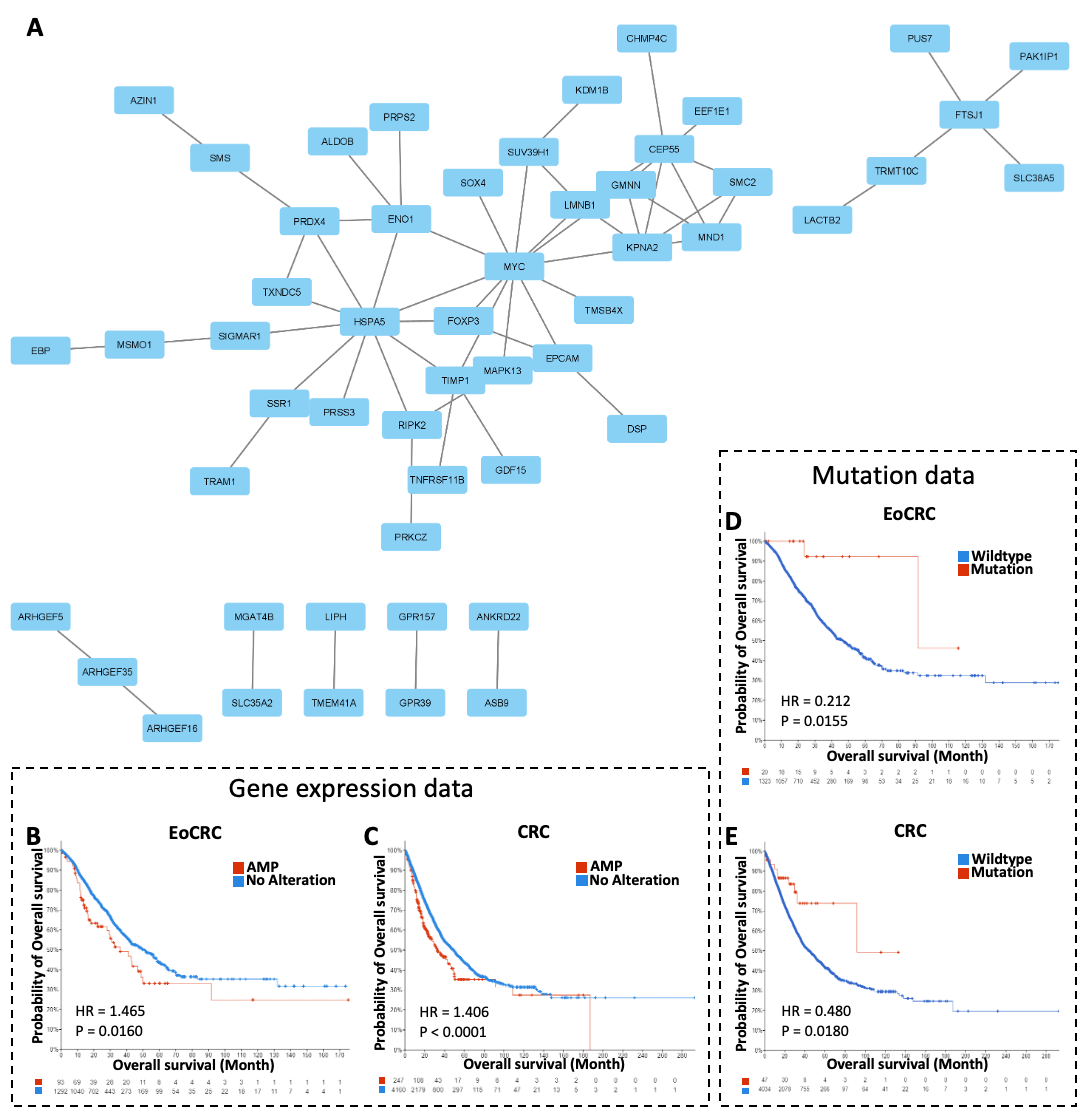


**Supplementary Figure 1.** Protein-protein interaction (PPI) of EoCRC overexpression genes. **(**A) Protein-protein interaction (PPI) map of EoCRC overexpression genes analyzed by STRING. (B, C) Comparison of overall survival between AMP and No Alteration hub genes in EoCRC and CRC cohort, respectively. (D, E) Comparison of overall survival between Wildtype and Mutation hub genes in EoCRC and CRC cohort, respectively.


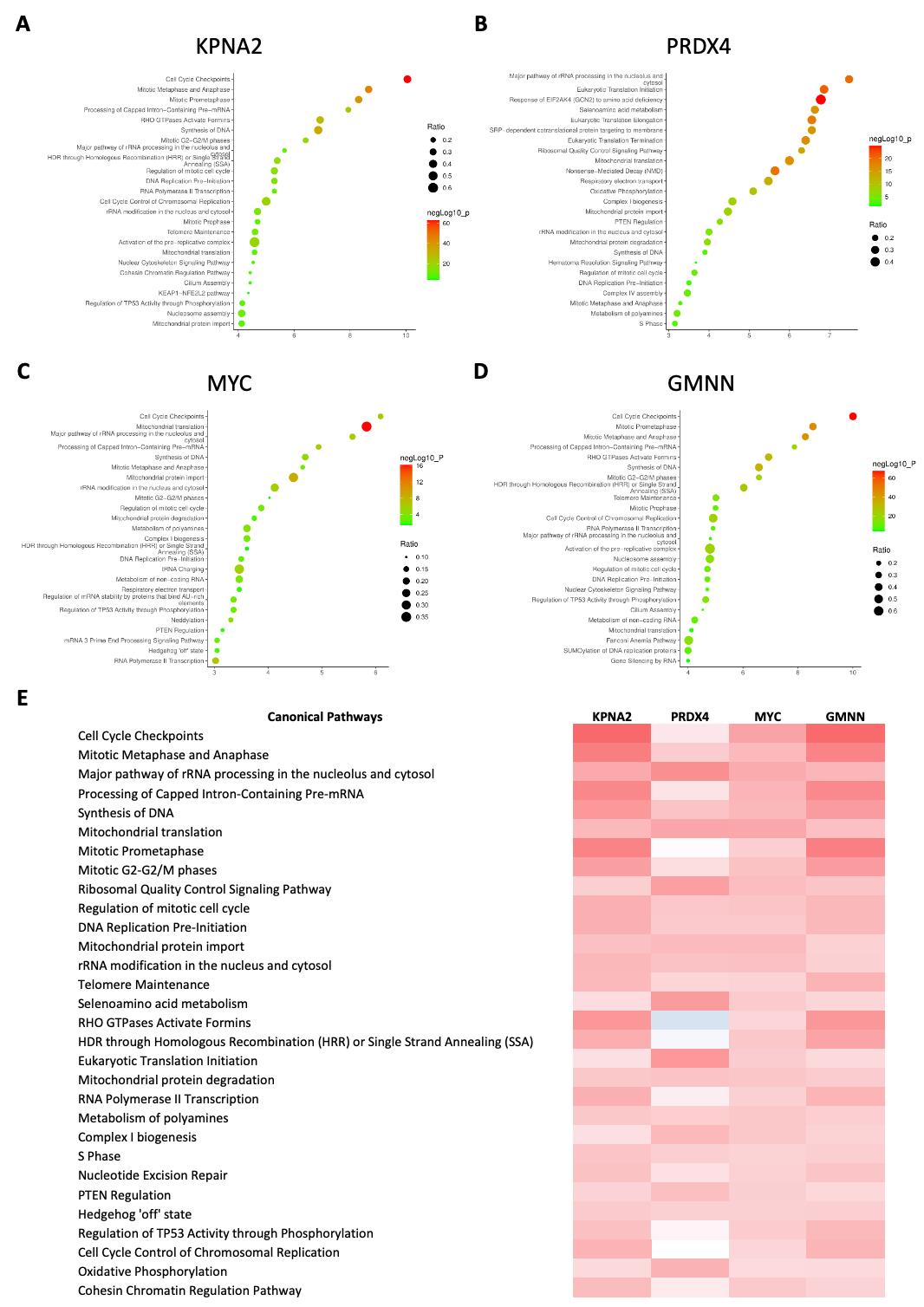


**Supplementary Figure 2.** Hub gene function pathway prediction and comparison analysis using correlation genes in TCGA EoCRC cohort. (A-D) Ingenuity Pathway Analysis (IPA) of four selected hub genes, illustrating their predicted functional pathways. (E) Pathway comparison analysis highlighting the shared biological pathways among these hub genes.


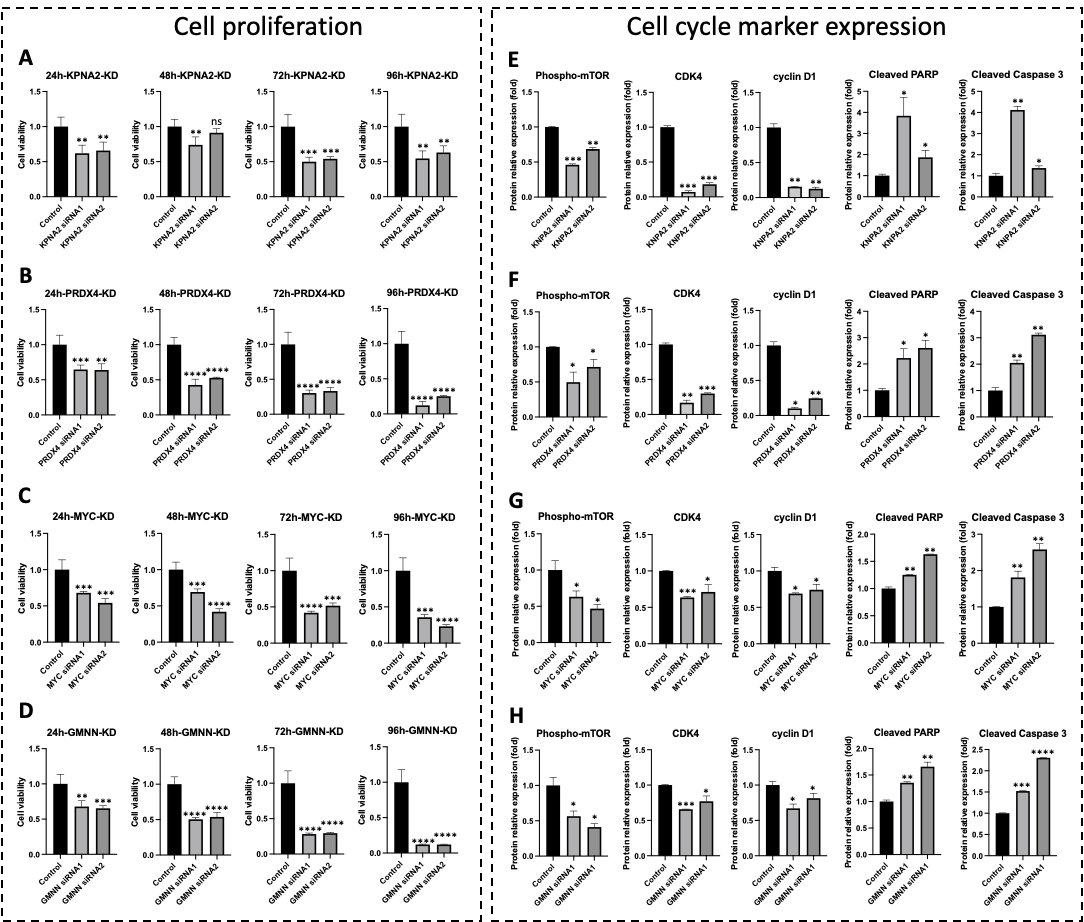


**Supplementary Figure 3.** Statistical analysis of the effect of Hub Genes on the cell proliferation and cell cycle marker of EoCRC cell line HCT116. ns: non-statistical significance. (A-D) Cell viability comparison between control and knockdown HCT116 cell lines, assessed every 24 hours after knockdown for each of the four hub genes. (E-H) Quantitative protein expression levels of cell cycle and cell death markers for each knockdown cell lines, analyzed using ImageJ software. ns: non-statistical significance; *: p≤0.05; **: p≤0.01; ***: p≤0.001; ****: p≤0.0001.
